# Strong catchment-specific structuring of Swedish wastewater microbiomes in a paired two-timepoint metagenomic survey

**DOI:** 10.3389/fmicb.2026.1907599

**Published:** 2026-07-29

**Authors:** Dhananjay Mukhedkar, Milan S. Stosic, Anna J. Székely, Ekaterina Avershina, Laila Sara Arroyo Mühr

**Affiliations:** 1 Center for Cervical Cancer Elimination, F56, Karolinska University Hospital Huddinge, Karolinska Institutet, Huddinge, Sweden; 2 Hopsworks AB, Stockholm, Sweden; 3 Norwegian HPV Reference Laboratory, Department of Microbiology and Infection Control, Akershus University Hospital, Lørenskog, Norway; 4 Department of Aquatic Sciences and Assessment, Science for Life Laboratory, Swedish University of Agricultural Sciences, Uppsala, Sweden; 5 Department of Tumor Biology, Institute of Cancer Research, Oslo University Hospital, Oslo, Norway; 6 Department of Pharmacy, Centre for Bioinformatics, University of Oslo, Oslo, Norway

**Keywords:** catchment-specific microbiome, influent wastewater, shotgun metagenomics, wastewater metagenomics, wastewater microbiome, wastewater-based surveillance

## Abstract

**Introduction:**

Wastewater microbial communities integrate signals from human populations, environmental inputs, and sewer infrastructure, but the extent to which these communities vary between wastewater catchments compared with individual sampling occasions remains incompletely understood.

**Methods:**

We analyzed influent wastewater from 16 wastewater treatment plant sites across Sweden, collected at two paired within-year timepoints, Week 3 and Week 21, using shotgun metagenomic sequencing and compositional data analysis.

**Results:**

Classified genus-level profiles were dominated by bacteria (96.54%), with smaller contributions from viruses (2.03%), eukaryota (1.05%) and archaea (0.40%). Genus-level alpha diversity increased between the two sampled timepoints, with median within-site changes of +10 genera in richness and +0.21 in Shannon diversity (*p* < 0.003). In contrast, overall community composition was primarily structured by wastewater treatment plant site: site explained 58.3% of total variance (*p* = 0.0003), whereas sampling timepoint explained 3.7% and was not significant (*p* = 0.106). Within-site compositional change between the two timepoints was nevertheless evident (*p* = 4.8 × 10^−4^), but the magnitude and direction of change varied across sites, indicating heterogeneous local shifts rather than a synchronized national temporal pattern. Genera detected in at least 75% of sites at both sampled timepoints accounted for most classified community abundance, whereas most measured within-site Aitchison turnover was accounted for by genera outside the high-prevalence shared fraction. Geographic distance and the number of connected inhabitants showed no significant association with genus-level community composition.

**Discussion:**

These findings indicate that Swedish influent wastewater microbiomes are strongly catchment-specific across paired sampling timepoints and support the use of site-specific reference profiles when interpreting wastewater metagenomic data. Denser temporal sampling and additional catchment metadata will be needed to assess seasonality, long-term stability, and the local drivers of wastewater microbiome variation.

## Introduction

Wastewater harbors highly diverse microbial communities that integrate inputs from human populations, industrial sources, sewer infrastructure, and the surrounding environment. These communities include gut-associated bacteria, environmental taxa, and opportunistic pathogens, making wastewater microbiomes a useful interface between human health, urban ecology, and environmental monitoring. In recent years, they have gained increasing attention both as ecological systems of interest and as a basis for wastewater-based surveillance, where microbial signals can be used to monitor population-level trends (Hendriksen et al., 2019; Newton et al., 2015; Bibby and Peccia, 2013).

Previous studies have described the taxonomic composition and functional potential of wastewater microbiomes (McLellan et al., 2015; Wu et al., 2019; Székely et al., 2025). These studies have consistently highlighted pronounced variation between wastewater systems, reflecting differences in local environmental conditions, sewer infrastructure, catchment characteristics, and population inputs (Fierer et al., 2022). At the same time, temporal changes have been reported, ranging from seasonal shifts to variation between sampling occasions (Ju and Zhang, 2015). However, the relative contribution of between-catchment differences compared with variation between individual sampling occasions remains incompletely characterized at national scale (Becsei et al., 2024). In particular, it remains unclear whether within-year temporal changes in wastewater microbiomes occur coherently across multiple sites or are largely heterogeneous and catchment-specific.

Another open question concerns how community-level change is distributed across taxa. Many microbial ecosystems include broadly shared, high-prevalence taxa together with more variable, lower-prevalence members that may contribute disproportionately to turnover and variability (Gonze et al., 2018; Shade and Handelsman, 2012). In the present study, however, “high prevalence” refers only to taxa broadly detected across sites at both sampled timepoints and does not imply a persistent or long-term stable core microbiome. Although this core–periphery framework has been widely discussed in host-associated and environmental microbiomes, its application to influent wastewater remains limited. Distinguishing changes in broadly detected taxa from shifts in lower-prevalence taxa is important for interpreting wastewater metagenomic data, particularly in surveillance contexts where reproducibility and comparability across sites are essential.

To address these questions, we analyzed influent wastewater microbiomes from 16 wastewater treatment plants across Sweden collected at two paired within-year timepoints, Week 3 and Week 21. Specifically, we asked: (i) whether genus-level alpha and beta diversity differed between the two sampled timepoints, (ii) how much community composition varied between wastewater catchments compared with within-year temporal variation across the two sampled timepoints, and (iii) whether within-site compositional turnover was mainly associated with broadly detected high-prevalence genera or with lower-prevalence taxa. We hypothesized that wastewater catchments would retain distinct genus-level community profiles across the two sampled timepoints, while temporal differences would be heterogeneous across sites and mainly involve lower-prevalence taxa.

## Materials and methods

### Sampling strategy and study design

The present study included influent wastewater samples collected from 16 wastewater treatment plants (WWTPs) across Sweden at two timepoints: Week 3 (W3) and Week 21 (W21) in 2024. These 2 weeks were selected because they were the only 2024 sampling rounds for which paired samples were available from all 16 sites included in the study. Other sampling weeks were available for subsets of sites but did not provide complete nationwide coverage. The selection was therefore based on completeness and comparability across sites rather than on a predefined seasonal contrast.

The study sites were Gävle (Duvbackens avloppsreningsverk), Göteborg (Ryaverket), Kalmar (Kalmarsundsverket), Karlstad (Sjöstadsverket), Linköping (Nykvarnsverket), Luleå (Uddebo reningsverk), Malmö (Sjölunda reningsverk), Umeå (Öns reningsverk), Uppsala (Kungsängsverket), Västerås (Kungsängsverket), Östersund (Gövikens reningsverk), Östhammar (Alunda avloppsreningsverk), and four locations in the Stockholm region: Bromma (Bromma reningsverk), Grödinge (Himmerfjärdsverket), Henriksdal (Henriksdals reningsverk), and Käppala (Käppalaverket). The geographic coordinates of all wastewater treatment plant sites are provided in Supplementary Table S1.

At each site, influent wastewater samples were obtained following standardized collection procedures. For most sites, untreated wastewater samples representative of a single day were collected using flow-compensated samplers at the WWTP. Uppsala was the exception, where daily samples were combined flow-proportionally into one composite weekly sample for analysis. All samples were transported under cooled conditions to the analysis laboratory immediately after collection and processed for nucleic acid extraction within 48 h of arrival. Exact holding times and transport temperatures were not recorded consistently across all sites and were therefore not analyzed as predictors in this study. Consequently, we cannot exclude that variation in pre-analytical conditions may have affected microbial persistence, growth, or decay before nucleic acid extraction.

### Extraction and sequencing

Total nucleic acids were extracted from 40 mL of influent wastewater using the Maxwell® RSC Enviro TNA Kit (Promega Corporation, Madison, WI, USA) on the Maxwell® RSC instrument (Promega Corporation, Madison, WI, USA), following the manufacturer’s instructions with the PureFood GMO and Authentication program, as described previously (Isaksson et al., 2022). Total genomic DNA was quantified using the Quant-iT™ PicoGreen™ dsDNA Assay Kit (Thermo Fisher Scientific, Waltham, MA, USA). Shotgun metagenomic sequencing of total DNA was performed using the Illumina DNA Prep Tagmentation kit (Illumina, Inc., San Diego, CA, USA) with dual indices, following the manufacturer’s instructions. Individual libraries were sequenced using paired-end sequencing (2 × 150 bp) on the NovaSeq 6,000 S4 platform (Illumina^®^), with a median output of 32.2 Gb per sample after quality trimming.

### Sequencing analysis

Raw reads were processed using Biopipe^1^, a workflow for standardized taxonomic classification of metagenomic data. The pipeline consists of two main steps: (i) bioinformatic preprocessing of sequencing data and (ii) taxonomic classification, followed by downstream analysis of diversity and community composition.

Briefly, adapter removal and quality trimming were performed using Trimmomatic (minimum read length: 36 bp; slidingWindow: 4:15; Bolger et al., 2014) and read quality was assessed before and after trimming with FastQC. High-quality reads were screened for human sequences using NextGenMap against the human reference genome GRCh38 (Sedlazeck et al., 2013), with non-default parameters of >95% identity over at least 75% of the read length. Reads classified as human were removed, and non-human reads were extracted using SAMtools and reconverted to FASTQ format. Taxonomic classification was performed using Kraken2 (Wood et al., 2019), v2.1.2, with a RefSeq database built in June 2024 and including archaea, bacteria, viruses, plasmids, protozoa, fungi, UniVec_Core, and two human references: GRCh38 and T2T-CHM13v2.0. Human libraries were created with the no-mask option. Bracken v2.9 was used on Kraken2 reports to refine abundance estimates to the genus level for downstream analyses (Lu et al., 2017). No technical sequencing replicates were generated; the study design focused on paired site comparisons across two timepoints.

To ensure computational efficiency, all analyses were executed on an on-premises Hopsworks cluster, version 3.1,^2^ running Apache Spark for distributed execution of the Biopipe workflow, enabling parallelized preprocessing and taxonomic classification.

### Downstream diversity analysis and statistics

Before prevalence or compositional calculations, fixed detection thresholds were applied to the classified genus-level profiles to reduce spurious low-abundance calls: taxa were retained if their relative abundance fraction was ≥0.001 and if the number of reads was ≥50. After filtering, genus-level relative abundance profiles were renormalized within each sample. Richness, Shannon diversity, prevalence, and presence/absence analyses were calculated from these filtered and renormalized genus-level profiles. Compositional analyses were performed using a centered log-ratio (CLR) transformation, with a small pseudocount (*ε* = 1 × 10^−6^) added before transformation to avoid undefined log values.

#### Diversity analyses

Genus-level richness and Shannon diversity indices (alpha diversity) were calculated for each sample using the filtered and renormalized relative abundance profiles to assess within-sample alpha diversity. Differences between W3 and W21 within each site were evaluated using paired Wilcoxon signed-rank tests.

Between-sample community structure (beta diversity) was assessed using Aitchison distances, calculated as Euclidean distances on CLR-transformed genus-level relative abundance profiles. The effects of site and sampling timepoint were analyzed using PERMANOVA with adonis2 and 9,999 permutations, reporting marginal by-term tests. A timepoint-only model was also fitted with site used as permutation strata to respect the paired study design. In parallel, a presence/absence matrix was constructed at the genus level after applying detection thresholds, and binary Jaccard distances were computed. The same PERMANOVA designs were applied to the Jaccard matrix. Principal coordinates analysis was applied to both Aitchison and Jaccard distances for visualization. Differences in community dispersion between W3 and W21 were evaluated using PERMDISP with betadisper and permutation tests for both distance types.

To directly evaluate whether paired samples retained site-associated similarity across the two sampled timepoints, each W21 sample was compared with its matched W3 counterpart and with the W3 samples from all other sites. For each site, the Aitchison distance between the matched W3–W21 pair was compared with the distribution of distances between that W21 sample and the W3 samples from all other sites. We recorded whether the matched W3 sample was the nearest W3 sample, among the three nearest, or closer than the median non-matched W3 distance. Matched distances were compared with the site-specific median non-matched distances using a paired one-sided Wilcoxon signed-rank test. Statistical significance was also assessed using a permutation test in which W3 site labels were randomly reassigned to generate the null distribution of matched-site distances.

#### Turnover and genus contributions

Within-site temporal turnover was quantified as the Aitchison distance between W3 and W21 CLR-transformed genus-level compositions for each site. The across-site distribution of turnover was summarized using the sample median and a nonparametric bootstrap 95% confidence interval based on 10,000 resamples.

For each site, ΔCLR was calculated for each genus as W21–W3. Squared ΔCLR values were summed across genera to obtain the squared Aitchison turnover for each site. Genera were ranked by their within-site contribution to turnover, and each genus’s mean turnover share was summarized across sites.

#### High-prevalence shared and lower-prevalence taxa

For this two-timepoint study, high-prevalence shared genera were defined as genera detected in at least 75% of sites at both sampled timepoints. This definition was used to distinguish broadly detected taxa from lower-prevalence taxa and was not intended to infer long-term temporal stability. For each site and timepoint, the fraction of classified community abundance explained by high-prevalence shared genera was calculated. Changes in this fraction between W3 and W21 were tested using paired Wilcoxon signed-rank tests, reporting the Hodges–Lehmann paired median difference and 95% confidence interval. The lower-prevalence fraction was defined as 1 minus the high-prevalence shared fraction. Within-site turnover was correlated with changes in the high-prevalence shared and lower-prevalence fractions using Spearman’s *ρ*.

#### Prevalence shifts

To test W3-to-W21 changes in genus prevalence across sites, paired 2 × 2 tables were constructed for each genus to count site-level gains (absent to present) and losses (present to absent). McNemar’s test was applied per genus, and *p*-values were adjusted using the Benjamini–Hochberg (BH) false discovery rate (FDR). Signed prevalence change was reported as Δ = (gains − losses)/number of sites, alongside FDR-adjusted *p*-values.

#### Concordance of community configurations

Timepoint-specific CLR principal component analysis scores, using up to five components, were calculated for the set of paired sites. Geometric concordance between W3 and W21 ordination configurations was quantified with Procrustes analysis and tested using PROTEST with 9,999 permutations.

#### Differential abundance

Differential abundance was evaluated using two complementary compositional frameworks. With ANCOM-BC2, genus-by-sample count matrices were used as input. To evaluate the global site effect adjusted for sampling timepoint, a fixed-effects model including site and timepoint was fitted. To evaluate the timepoint effect, a mixed-effects model with a random intercept for site was fitted. Genera observed in fewer than two distinct sites were excluded to satisfy model identifiability. Multiple testing was controlled using the BH FDR.

In ALDEx2, a paired genus-level W3 versus W21 analysis was performed using Dirichlet-Monte Carlo centered log-ratio transformation with 128 Monte Carlo samples. Multiple testing was again controlled using the BH FDR, and ALDEx2 effect sizes were reported in centered log-ratio units. The ANCOM-BC2 mixed-effects model was considered the primary timepoint analysis because it explicitly modeled site as a random intercept, while the paired ALDEx2 analysis was used as a sensitivity analysis.

#### Geographic distance and the number of connected-inhabitant effects

Geographic distance effects were assessed by correlating pairwise Aitchison dissimilarities with great-circle geographic distances in kilometers using Mantel tests with Spearman correlations and 9,999 permutations. To compare distance–decay patterns between W3 and W21, a paired permutation procedure was used in which timepoint labels were shuffled within site pairs and the difference in regression slopes was tested. Multi-scale spatial signal was further evaluated by deriving principal coordinates of neighbor matrices (PCNM) from the geographic distance matrix and fitting distance-based redundancy analyses of Aitchison dissimilarities on the subset of positive-eigenvalue PCNM axes, up to five axes, separately for each timepoint. Significance was assessed using permutation analysis of variance, and adjusted *R*^2^ was reported.

Each site was linked to the number of connected inhabitants, retrieved from previous studies (Persson et al., 2025). The number of connected inhabitants was analyzed on the log_10_ scale. Associations with community features were evaluated in three ways: (i) for alpha diversity, ordinary least squares models of richness and Shannon diversity were fitted on log_10_-connected inhabitants separately by timepoint, and paired changes in alpha diversity (W21–W3) were regressed on log_10_-connected inhabitants, reporting Spearman’s *ρ* alongside the ordinary least squares slope and *p*-value; (ii) for temporal turnover, within-site Aitchison distance between W3 and W21 was related to log_10_-connected inhabitants using linear regression and Spearman correlation; and (iii) at the dissimilarity level, Aitchison distances were compared with pairwise absolute differences in log-connected inhabitants using Mantel and partial Mantel tests controlling for geographic distance, and distance-based redundancy analysis models of Aitchison dissimilarities on log_10_-connected inhabitants were fitted, reporting permutation *p*-values and adjusted *R*^2^.

#### Permutation, resampling, and multiple testing

Unless otherwise noted, permutation tests used 9,999 permutations, with pairing respected where appropriate by using site as strata. Bootstrap confidence intervals for medians were generated with 10,000 resamples. Multiple tests across genera were controlled using the BH FDR.

### Ethics statement

This study was based exclusively on pooled influent wastewater samples collected at wastewater treatment plants. It did not involve intervention, contact with individuals, individual-level data, or identifiable human material. Therefore, ethics approval for human-subject research was not required.

## Results

### Dataset overview

Across 32 samples, representing 16 wastewater treatment plant sites sampled at two timepoints, shotgun metagenomic sequencing yielded a median of 50.0 million high-quality read pairs per sample. After preprocessing and removal of reads classified as human, Kraken2 assigned a median of 24.9 million reads per sample to bacteria, viruses, or archaea. Among classified reads, profiles were dominated by bacteria, with a median relative abundance of 96.54%, followed by viruses at 2.03%, eukaryota at 1.05%, and archaea at 0.40%. Fungal and protozoan assignments were included within the eukaryotic fraction. Protozoa were not summarized as a separate aggregate category because they do not constitute a single taxonomic rank in the NCBI taxonomy.

### Alpha diversity

**Figure 1:**
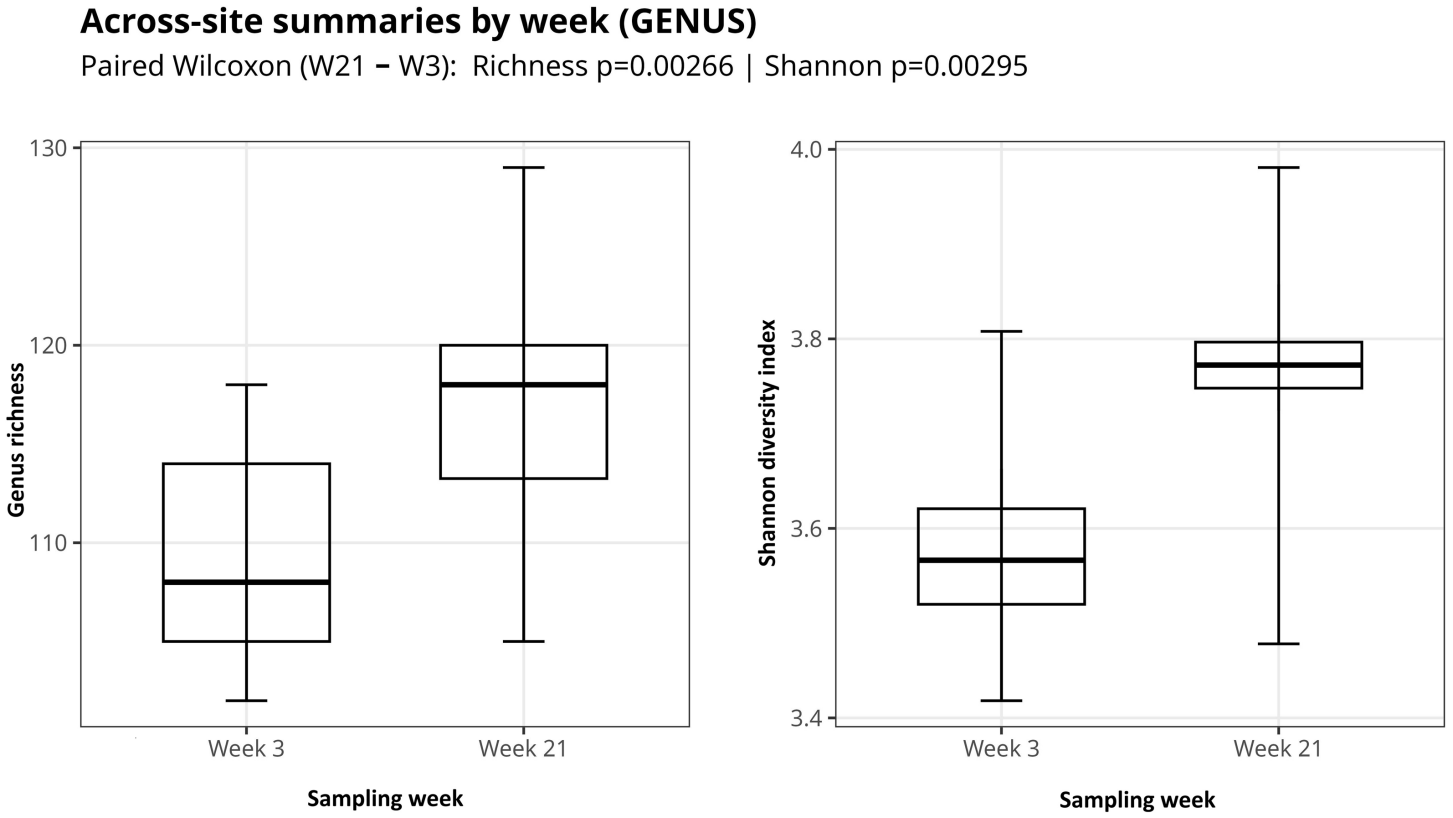
Genus-level alpha diversity at W3 and W21 across 16 wastewater treatment plant sites. Boxplots show the distributions of genus-level richness and Shannon diversity at week 3 (W3) and week 21 (W21). Boxes indicate the median and interquartile range, and whiskers indicate the range. Differences between timepoints were assessed using paired Wilcoxon signed-rank tests.

Across the 16 paired wastewater treatment plant sites, genus-level alpha diversity significantly increased from Week 3 to Week 21. Median within-site change was +10 genera for richness and +0.209 for Shannon diversity (paired Wilcoxon W21–W3: richness *p* = 0.00266, Shannon *p* = 0.00295, *n* = 16). The direction of change was broadly consistent across sites, although the magnitude varied by site (Figures 1A,B).

### Beta diversity

Community composition differed primarily by wastewater treatment plant site, with little consistent change between the two sampled timepoints. In PERMANOVA on Aitchison distances, site explained 58.3% of variance (*R*^2^ = 0.583, *p* = 0.0003), whereas sampling timepoint accounted for 3.7% and was not significant (*R*^2^ = 0.037, *p* = 0.106). Because each site was represented by only two paired samples, the site term was interpreted cautiously as evidence of catchment-associated structuring within the present paired dataset rather than as a fully powered estimate of a generalizable site effect. A Bray–Curtis sensitivity analysis showed the same pattern (site *R*^2^ = 0.651, *p* = 0.0017; timepoint *R*^2^ = 0.047, *p* = 0.0609). When the timepoint effect was tested within pairs, using site as permutation strata, it remained non-significant for Aitchison distances (*p* = 0.0878).

**Figure 2:**
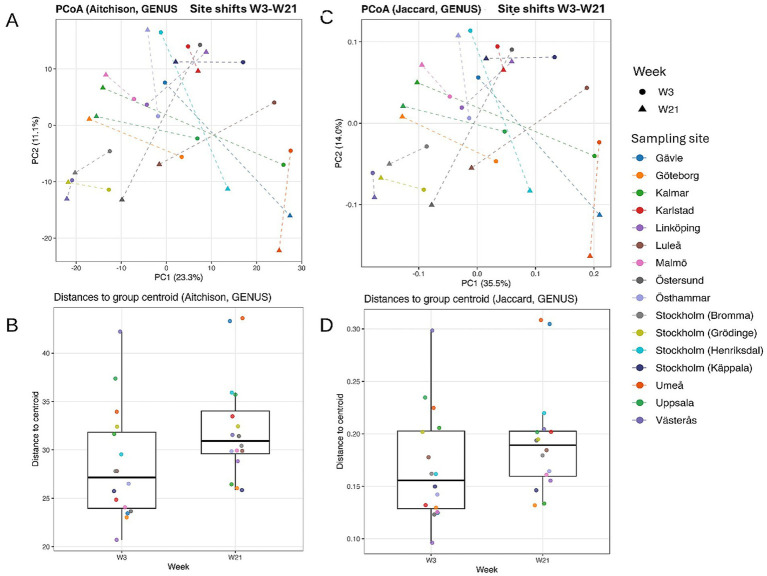
Beta diversity in wastewater microbiomes across 16 Swedish wastewater treatment plant sites. **(A,B)** Principal coordinate analysis (PCoA) and dispersion of genus-level community profiles based on Aitchison distances. **(A)** PCoA showing paired W3 and W21 samples connected by dashed lines. Colors denote sampling sites; circles indicate W3 and triangles indicate W21. Each paired line represents the within-site shift between the two sampled timepoints. **(B)** Community dispersion, expressed as distance to the timepoint centroid in Aitchison space. **(C,D)** PCoA and dispersion based on presence/absence structure using Jaccard distances. **(C)** PCoA showing paired W3 and W21 samples connected by dashed lines, as in panel **(A)**. **(D)** Community dispersion, expressed as distance to the timepoint centroid in Jaccard space.

Principal coordinate analysis was used to visualize beta-diversity structure rather than as the primary evidence for site-associated similarity. The ordinations showed no coherent separation by sampling timepoint and no synchronized national W3-to-W21 shift. Instead, paired site shifts varied in both direction and magnitude, consistent with heterogeneous local temporal changes (Figures 2A,C). Site-associated similarity was therefore evaluated directly using pairwise Aitchison distances. Each W21 sample was compared with its matched W3 counterpart and with the W3 samples from all other sites. The matched W3 sample was the nearest W3 sample for five of 16 sites and among the three nearest W3 samples for six of 16 sites. More generally, W21 samples were closer to their own W3 counterpart than to the site-specific median non-matched W3 distance in 12 of 16 sites. The median matched W3–W21 Aitchison distance was 37.6, compared with 44.5 for the median non-matched W3 distance. Matched distances were lower than the site-specific median non-matched distances (paired Wilcoxon signed-rank test, *p* = 0.0181), and a site-label permutation test indicated that matched-site distances were lower than expected by chance (*p* = 1.0 × 10^−4^). Thus, paired samples showed measurable temporal turnover but nevertheless tended to retain greater similarity to their own site than to unrelated sites.

Group dispersion changed only modestly. Distances to the timepoint centroid were higher at W21 than W3 for Aitchison distances (median 30.9 vs. 27.2), but the PERMDISP test was borderline (*F* = 3.61, *p* = 0.066; Figure 2B). For Jaccard distances, dispersion did not differ significantly between timepoints (*F* = 1.80, *p* = 0.19; Figure 2D). Overall, dispersion differences between W3 and W21 were limited and did not indicate a coherent nationwide temporal shift.

### Turnover

**Figure 3:**
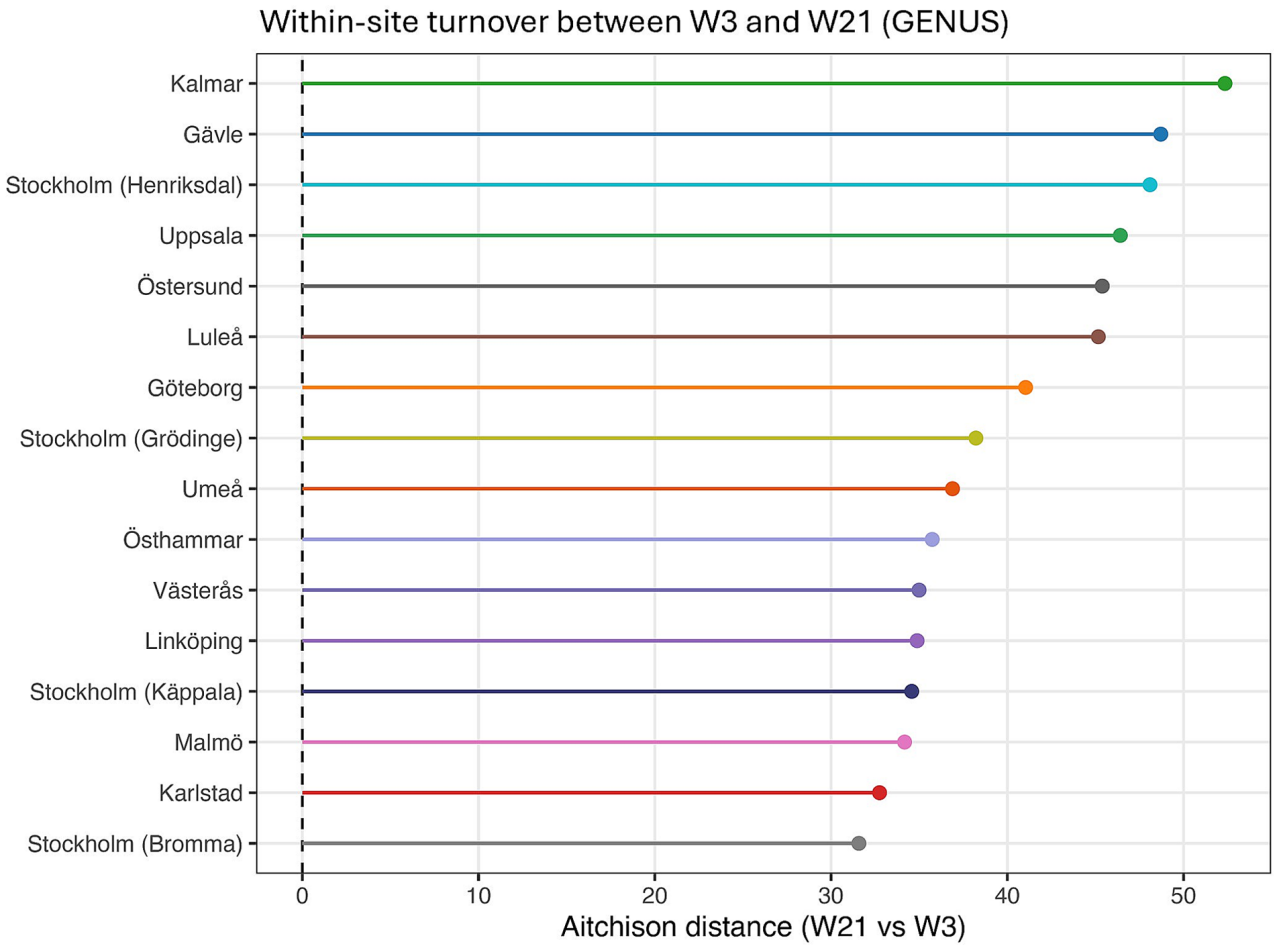
Per-location microbial turnover between W3 and W21. Aitchison distance between W3 and W21 CLR-transformed genus-level profiles for each sampling site. Larger values indicate within-site compositional change between the two sampled timepoints. The dashed vertical line marks the zero reference.

Within-site community composition changed between W3 and W21. Turnover, quantified as the Aitchison distance between the two timepoints’ CLR-transformed genus-level profiles, had a median of 37.5 across sites (bootstrap 95% CI 34.9–45.3). Magnitudes varied across sites, ranging from 31.5 to 52.3, indicating measurable but heterogeneous within-site compositional change between the two sampled timepoints (Figure 3).

### High-prevalence shared and lower-prevalence fractions

High-prevalence shared genera were defined as genera detected in at least 75% of sites at both sampled timepoints. This group included 88 genera, and the full list of genera with per-timepoint prevalence and mean abundance is shown in Supplementary Table S2.

For each site, the fraction of total classified relative abundance explained by high-prevalence shared genera was computed at W3 and W21. This fraction decreased from W3 to W21 (Hodges–Lehmann paired median Δ = −0.020; 95% CI −0.0312 to −0.0103; *p* = 0.00209), indicating a small proportional increase in the lower-prevalence fraction (Supplementary Figure S1).

**Figure 4:**
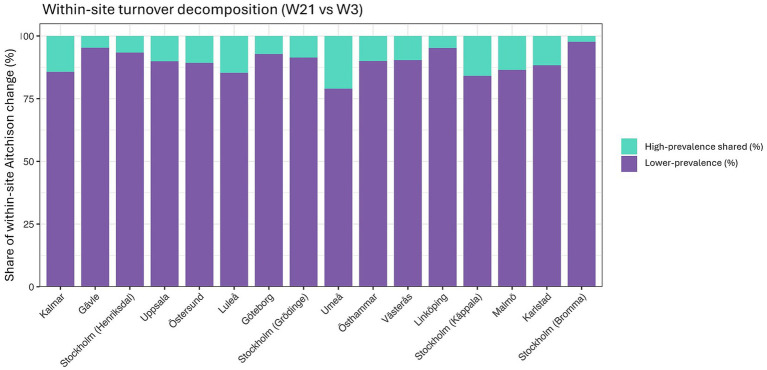
Within-site microbial turnover decomposition between W3 and W21. Proportion of measured within-site Aitchison turnover accounted for by high-prevalence shared genera, defined as genera detected in at least 75% of sites at both sampled timepoints, and by lower-prevalence genera, defined as all remaining genera. Bars are stacked to 100% for each sampling site. This decomposition is descriptive and should not be interpreted as evidence that lower-prevalence genera biologically caused turnover.

Within-site compositional turnover was not associated with changes in the high-prevalence shared fraction. Sites exhibiting greater W3-to-W21 turnover did not systematically lose or gain abundance in this fraction (Spearman’s *ρ* = 0.10, *p* = 0.69). A per-site decomposition of turnover into high-prevalence shared and lower-prevalence components is provided in Supplementary Table S3. Across sites, most within-site turnover between W3 and W21 was attributable to the lower-prevalence fraction rather than to high-prevalence shared genera (Figure 4), consistent with the decomposition results.

### Presence/absence and differential abundance

Three complementary signals were evaluated: (i) between-site differences in genus abundance using the ANCOM-BC2 global site effect adjusted for sampling timepoint, (ii) within-site differences between W3 and W21 using an ANCOM-BC2 mixed model with a random intercept for site and paired ALDEx2 tests, and (iii) changes in genus presence/absence between W3 and W21 using paired McNemar tests.

ANCOM-BC2 identified a substantial site effect: 86 of 225 genera (38.2%) were significant for the global site term after FDR correction (*q* < 0.05; Supplementary Table S4). Significant taxa included gut-associated genera, such as *Bacteroides*, *Faecalibacterium*, and *Bifidobacterium*, as well as wastewater- or environment-associated genera, such as *Pseudomonas*, *Comamonas*, and *Polaromonas*. This was consistent with the strong site-associated differences observed in the beta-diversity analyses.

Using the mixed-effects ANCOM-BC2 model for the timepoint effect, only *Butyricimonas* showed a significant W21–W3 shift after FDR correction (*q* = 0.0478; log-fold change = −0.42, indicating lower abundance at W21). The paired ALDEx2 sensitivity analysis identified 47 of 225 genera (20.9%) after FDR correction (Supplementary Table S5), but *Butyricimonas* was not among them. Thus, no genus was significant in both analyses. We therefore considered genus-level timepoint signals method-dependent and did not use individual differential-abundance findings as primary evidence for temporal change.

Paired McNemar tests found no genera with FDR-significant prevalence changes between W3 and W21 (all *q* = 1; *n* = 16).

### Geographic distance and the number of connected inhabitants size do not explain between-site community differences

To test whether geographic distance was associated with wastewater microbiome composition, Mantel tests were performed between pairwise Aitchison dissimilarities and inter-site geographic distances. No significant association was detected at either timepoint (W3: *r* = −0.109, *p* = 0.716; W21: *r* = 0.021, *p* = 0.414). Distance–decay slopes were shallow and did not differ significantly between timepoints (W3 slope = −0.003; W21 slope = +0.00217; ΔSlope = 0.00516, *p* = 0.197; Supplementary Figure S2). PCNM-based dbRDA detected no significant spatial structuring (W3 adjusted *R*^2^ = −0.0016, *p* = 0.489; W21 adjusted *R*^2^ = 0.00512, *p* = 0.430), and Procrustes/PROTEST analyses indicated weak geographic–microbial concordance (W3 *p* = 0.136; W21 *p* = 0.0454). The number of connected inhabitants was also not associated with genus-level diversity or community composition. In models using log_10_-connected inhabitants, alpha diversity was not associated with the number of connected inhabitants at either timepoint (OLS *p* = 0.81; Spearman’s *ρ* = 0.173, *p* = 0.522). Paired W21–W3 changes in alpha diversity were also unrelated to the number of connected inhabitants (richness: slope = 0.347, *p* = 0.916; Spearman’s *ρ* = −0.065, *p* = 0.811; Shannon: slope = 0.0013, *p* = 0.985; Spearman’s *ρ* = −0.193, *p* = 0.474). Similarly, within-site Aitchison turnover was not associated with the number of connected inhabitants (slope = −0.511, *p* = 0.873; Spearman’s *ρ* = −0.21, *p* = 0.434). At the dissimilarity level, Mantel and partial Mantel tests were non-significant at both W3 and W21, and dbRDA models showed no explanatory power for the number of connected inhabitants (W3 adjusted *R*^2^ = −0.0145, *p* = 0.673; W21 adjusted *R*^2^ = −0.000147, *p* = 0.434; Supplementary Table S6).

## Discussion

This paired two-timepoint field study evaluated how wastewater treatment plant site and within-year temporal variation shape influent wastewater microbiomes across Sweden. The main finding was that community composition showed strong catchment-associated structuring within this paired dataset, whereas sampling timepoint explained comparatively little variation. PERMANOVA attributed far more variance to site than to timepoint, and ordination analyses did not reveal a coherent nationwide temporal pattern. Direct matched-site distance analyses further showed that, despite measurable temporal turnover, paired samples generally retained greater similarity to their own site than to samples from other sites. Together, these findings support the importance of site-specific interpretation in wastewater metagenomic surveillance, supporting the importance of site-specific interpretation in wastewater metagenomic surveillance. Temporal change was nevertheless evident. Genus-level alpha diversity increased from W3 to W21, and within-site Aitchison turnover showed measurable compositional change between the two sampled timepoints. However, the magnitude and direction of these shifts varied across sites, indicating heterogeneous local changes rather than a synchronized national temporal trend. This pattern suggests that paired within-year changes in influent wastewater microbiomes may reflect local catchment-specific dynamics rather than a uniform temporal signal across Sweden.

The distribution of measured turnover across taxa further supports a distinction between broadly detected and more variably detected genera. Genera detected in at least 75% of sites at both sampled timepoints accounted for a large fraction of classified community abundance, whereas most CLR-based W3-to-W21 turnover was accounted for by genera outside the high-prevalence shared fraction. This pattern is not unexpected, because the lower-prevalence category includes genera with intermittent detection by definition. This should be interpreted as an operational decomposition of observed compositional change rather than evidence that lower-prevalence taxa biologically caused temporal turnover. The result indicates that broadly detected genera contributed less to the measured Aitchison turnover than genera with more restricted or variable occurrence across sites. Importantly, because this study included only two timepoints, these high-prevalence shared genera should not be interpreted as a long-term stable core. Rather, they represent a broadly detected fraction within the temporal scope of the present study.

Differential-abundance analyses were consistent with a dominant site effect and weaker timepoint-associated signals. ANCOM-BC2 identified widespread between-site differences in genus abundance but detected only one genus with a significant W21-to-W3 shift after false discovery rate correction. In contrast, paired ALDEx2 identified a larger set of timepoint-associated genera. Because no genus was significant in both analyses, the timepoint-associated differential-abundance signals were not robust across statistical frameworks. We therefore treated taxon-level results as secondary and based the interpretation of temporal change primarily on community-level analyses.

Geographic distance did not appear to be a major driver of between-site wastewater microbiome differences. Mantel tests and distance–decay analyses showed no significant association between inter-site distance and Aitchison dissimilarity, and PCNM-based dbRDA did not detect significant spatial structuring. Although Procrustes/PROTEST analysis suggested weak geographic–microbial concordance at W21, this signal was not supported consistently across complementary analyses. Together, these findings suggest that the site effect is unlikely to reflect simple geographic distance or the number of connected inhabitants alone. Instead, it may arise from local catchment characteristics, sewer infrastructure, environmental inputs, industrial contributions, hydrology, and other unmeasured site-specific factors.

These findings have practical implications for wastewater-based monitoring. Cross-site comparisons of wastewater metagenomic data should account for strong site-associated differences, even when samples are collected within the same national sampling framework. Site-specific reference profiles may help distinguish persistent local community characteristics from temporal fluctuations, particularly when interpreting metagenomic surveillance signals. However, the present data should be viewed as a paired two-timepoint reference rather than a full temporal baseline. Establishing robust baselines for surveillance will require denser temporal sampling, standardized catchment metadata, and repeated sampling across seasons and years.

The findings align with previous studies showing that local wastewater system characteristics can outweigh temporal variation in microbial community composition. Prior work in sewer networks and wastewater treatment plants has reported site-specific microbial profiles and asynchronous temporal shifts, consistent with the present observation that site effects dominate over coordinated temporal change (Fierer et al., 2022; Wei et al., 2018). Our results also parallel studies of activated sludge in which broadly shared abundant taxa coexist with more variable lower-prevalence members (Saunders et al., 2016). However, differences in matrix, sequencing approach, sampling design, and temporal depth should be considered when comparing these studies. In the present influent wastewater dataset, the high-prevalence shared fraction and lower-prevalence fraction provide a useful operational framework for describing turnover, but long-term stability cannot be inferred from two timepoints.

A key limitation is the paired two-timepoint design, which cannot test seasonality or long-term temporal dynamics. W3 and W21 capture two within-year sampling occasions, but they do not allow separation of seasonal effects from other time-dependent or event-specific influences. In addition, detailed operational and physicochemical metadata from wastewater treatment plants, including chemical oxygen demand, total nitrogen, wastewater temperature, conductivity, industrial contributions, rainfall-related dilution, flow, and sewer-system configuration, were not consistently available across all sites and therefore could not be included as predictors. Although some parameters are measured by individual WWTP operators, retrospective harmonization across multiple plants is difficult because of differences in monitoring systems and data accessibility. These unmeasured operational and infrastructure-related factors may contribute to the observed site-associated differences. Future studies would benefit from systematic integration of operational metadata, physicochemical measurements, wastewater flow, rainfall data, sewer-network characteristics, and indicators of human fecal input such as pepper mild mottle virus concentrations.

Methodological constraints also remain. K-mer-based classifiers such as Kraken2 can be affected by misclassification, incomplete reference databases, and incomplete taxonomic recovery in complex metagenomes. Accordingly, the diversity and compositional analyses presented here should be interpreted as analyses of classified genus-level profiles rather than exhaustive descriptions of total wastewater microbial diversity. Future work should combine improved and better-curated reference databases with complementary assembly-based or marker-based approaches such as *de novo* metagenomic assembly, metagenome-assembled genome recovery, protein-level classification, marker-based profiling, and reference independent discovery methods to improve the detection of poorly represented or novel organisms. Such methodological advances, together with denser temporal sampling and richer catchment metadata, will be important for refining the interpretation of wastewater microbiome dynamics in surveillance and environmental monitoring. In conclusion, influent wastewater microbiomes across Sweden showed strong site-associated structuring across two paired within-year sampling timepoints. Differences between W3 and W21 were measurable but heterogeneous across sites, and most within-site turnover was attributable to lower-prevalence genera rather than to broadly detected high-prevalence genera. These findings support the use of site-specific reference profiles when interpreting wastewater metagenomic data, while emphasizing that denser temporal sampling and additional catchment metadata are needed to assess seasonality, long-term stability, and the drivers of local wastewater microbiome variation.

## Data Availability

The datasets supporting the conclusions of this article are available in the NCBI BioProject repository under accession number PRJNA1354877 (https://www.ncbi.nlm.nih.gov/sra/PRJNA1354877). All code used for processing and analysis of sequencing data is publicly available on GitHub: https://github.com/hpvcenter/biopipe and https://github.com/mim86/WW_stat_analysis.

## Footnotes

1
https://github.com/hpvcenter/biopipe

2
https://hopsworks.ai
