# Supplementary material for: Strong catchment-specific structuring of Swedish wastewater microbiomes in a paired two-timepoint metagenomic survey
Source: Front Microbiol. 2026 Jul 29;17:1907599. doi: 10.3389/fmicb.2026.1907599 (PMC13462427; doi:10.3389/fmicb.2026.1907599)
Supplement: Supplementary file 1 [file Data_Sheet_1.zip › Supplementary Data Sheet 1.DOCX]

**Supplementary Information**

for the article: Strong catchment-specific structuring of Swedish wastewater microbiomes in a paired two-timepoint metagenomic survey. Mukhedkar D, et al.

**Note**: This file contains Supplementary Figures S1–S2 and Supplementary Table S1, S3 and S6 associated with the main text. Supplementary Tables S2, S4 and S5 are provided as separate additional files in CSV format due to their large size.

**Table of Contents**

- **Supplementary Figure S1** – High-prevalence shared fraction by site	p. 2
- **Supplementary Figure S2** – Geographic distance–decay of wastewater microbiomes	p. 3
- **Supplementary Table S1 -** Geographic coordinates of the wastewater

treatment plant sites included in the study.	p. 4

- **Supplementary Table S3** – Within-site turnover decomposition	p. 5
- **Supplementary Table S6** – Associations between the number of connected inhabitants and genus-level diversity and community composition.	p. 6

**Supplementary Figure S1:** High-prevalence shared fraction by site

Mean fraction of total classified relative abundance explained by high-prevalence shared genera at Week 3 and Week 21 for each sampling site. High-prevalence shared genera were defined as genera detected in at least 75% of sites at both sampled timepoints.

**Supplementary Figure S2:** Geographic distance–decay of wastewater microbiomes at two sampled timepoints.

Scatterplots showing the relationship between geographic distance (x-axis) and Aitchison distance (y-axis) among wastewater treatment plants across Sweden at Week 3 (right) and Week 21 (left). Each point represents a pairwise comparison between sites. Shaded areas indicate 95% confidence intervals from linear regression.

**Supplementary Table S1**: Geographic coordinates of the wastewater treatment plant sites included in the study.

| **site** | **lat** | **lon** |
| --- | --- | --- |
| **Gavle** | 60.674553 | 17.141739 |
| **Goteborg** | 57.707500 | 11.967500 |
| **Kalmar** | 56.672681 | 16.361631 |
| **Karlstad** | 59.379300 | 13.503569 |
| **Linkoping** | 58.410861 | 15.621570 |
| **Lulea** | 65.584816 | 22.156704 |
| **Malmo** | 55.605293 | 13.000157 |
| **Stockholm_Bromma** | 59.336667 | 17.931111 |
| **Stockholm_Grodinge** | 59.067980 | 17.707182 |
| **Stockholm_Henriksdal** | 59.310110 | 18.109140 |
| **Stockholm_Kappala** | 59.355930 | 18.226410 |
| **Umea** | 63.825848 | 20.263035 |
| **Uppsala** | 59.858819 | 17.638889 |
| **Vasteras** | 59.611366 | 16.545025 |
| **Ostersund** | 63.179200 | 14.635661 |
| **Osthammar** | 60.259089 | 18.374081 |

**Supplementary Table S3**: Decomposition of within-site temporal turnover into high-prevalence shared and lower-prevalence contributions.

| Site | Turnover  (W21 vs W3, Aitchison) | High-prevalence shared turnover | Lower-prevalence turnover | High-prevalence shared share (%) | Lower-prevalence share (%) |
| --- | --- | --- | --- | --- | --- |
| Kalmar | 52.3 | 19.8 | 48.4 | 14.3 | 85.7 |
| Gävle | 48.7 | 10.5 | 47.6 | 4.7 | 95.3 |
| Stockholm (Henriksdal) | 48.1 | 12.3 | 46.5 | 6.6 | 93.4 |
| Uppsala | 46.4 | 14.8 | 44 | 10.1 | 89.9 |
| Östersund | 45.4 | 14.8 | 42.9 | 10.7 | 89.3 |
| Luleå | 45.2 | 17.3 | 41.7 | 14.7 | 85.3 |
| Göteborg | 41 | 11 | 39.5 | 7.2 | 92.8 |
| Stockholm (Grödinge) | 38.2 | 11.2 | 36.5 | 8.6 | 91.4 |
| Umeå | 36.9 | 16.9 | 32.8 | 21 | 79 |
| Östhammar | 35.7 | 11.3 | 33.9 | 10 | 90 |
| Västerås | 35 | 10.8 | 33.3 | 9.6 | 90.4 |
| Linköping | 34.9 | 7.7 | 34 | 4.8 | 95.2 |
| Stockholm (Käppala) | 34.6 | 13.8 | 31.7 | 15.9 | 84.1 |
| Malmö | 34.2 | 12.5 | 31.8 | 13.5 | 86.5 |
| Karlstad | 32.7 | 11.2 | 30.8 | 11.7 | 88.3 |
| Stockholm (Bromma) | 31.6 | 4.8 | 31.2 | 2.3 | 97.7 |

**Supplementary Table S6:** Associations between the number of connected inhabitants and genus-level diversity and community composition.

| **Analysis** | **Model/test** | **Estimate** | **p-value** |
| --- | --- | --- | --- |
| Alpha diversity vs log₁₀ connected inhabitants | OLS | – | 0.81 |
|  | Spearman | ρ = 0.173 | 0.522 |
| Δ Richness vs log₁₀ connected inhabitants | OLS | slope = 0.347 | 0.916 |
|  | Spearman | ρ = −0.065 | 0.811 |
| Δ Shannon diversity vs log₁₀ connected inhabitants | OLS | slope = 0.0013 | 0.985 |
|  | Spearman | ρ = −0.193 | 0.474 |
| Aitchison turnover vs log₁₀ connected inhabitants | OLS | slope = −0.511 | 0.873 |
|  | Spearman | ρ = −0.210 | 0.434 |
| Aitchison dissimilarity vs difference in connected inhabitants (W3) | Mantel | r = −0.0503 | 0.586 |
|  | Partial Mantel | r = −0.0524 | 0.595 |
| Aitchison dissimilarity vs difference in connected inhabitants (W21) | Mantel | r = −0.0660 | 0.666 |
|  | Partial Mantel | r = −0.0657 | 0.668 |
| Aitchison composition vs log₁₀ connected inhabitants (W3) | dbRDA | adj R² = −0.0145 | 0.673 |
| Aitchison composition vs log₁₀ connected inhabitants (W21) | dbRDA | adj R² = −0.000147 | 0.434 |

Ordinary least squares (OLS), Spearman correlation, Mantel, partial Mantel, and distance-based redundancy analysis (dbRDA) were used to evaluate associations between the number of connected inhabitants (log₁₀ scale) and alpha diversity, temporal turnover, pairwise community dissimilarity, and genus-level community composition. No statistically significant associations were observed in any analysis.
